# Supplementary material for: Rapid in situ quantification of the strobilurin resistance mutation G143A in the wheat pathogen Blumeria graminis f. sp. tritici
Source: Sci Rep. 2021 Feb 25;11:4526. doi: 10.1038/s41598-021-83981-9 (PMC7907364; doi:10.1038/s41598-021-83981-9)
Supplement: Supplementary file 1 — Supplementary Information. [file 41598_2021_83981_MOESM1_ESM.docx]

Rapid *in situ* quantification of the strobilurin resistance mutation G143A in the wheat pathogen Blumeria graminis f. sp. tritici

**Kejal N Dodhia^1^, Belinda A Cox^2^, Richard P Oliver^3^ and Francisco J Lopez-Ruiz^1^**^*^

^1^Centre for Crop and Disease Management, School of Molecular and Life Sciences, Curtin University, Perth, WA 6102, Australia

^2^Faculty of Science and Engineering, Curtin University, Perth, WA 6102, Australia

^3^Curtin University, Perth, WA 6102, Australia

^*^*Correspondence to: FJ Lopez-Ruiz, Centre for Crop and Disease Management, Curtin University, 6102 Bentley, Western Australia, Australia.*

*Email: fran.lopezruiz@curtin.edu.au*

**Supplementary Figure S1.**Linear correlation between percent of A143 allele in the mixture of DNA extracted from wheat leaves infected with *Blumeria graminis* f. sp. *tritici* mutant (A143) and wild (G143) type, quantified by digital PCR (R^2^ = 0.9988) and ASqPCR (R^2^ = 0.9994), and the input percentage of the mutant in a genomic DNA mixture of A143 and G143. Each point represents the average of triplicates. Error bars represent the s.e.m.

**Supplementary Table S2.** Area (ha) of wheat grown in different regions of Tasmania

| year | Cradle Coast (North West) | North (North Midlands) |
| --- | --- | --- |
| 2014-2015 | 1,186 | 6,165 |
| 2015-2016 | 977 | 7,360 |
| 2016-2017 | 1,046 | 7,953 |
| 2017-2018 | - | 5,167 |
| 2018-2019 | 881 | - |
| Average | 1023 | 6661 |

- represents unavailable data
